# Prevalence of Dementia and Subtypes in Valladolid, Northwestern Spain: The DEMINVALL Study

**DOI:** 10.1371/journal.pone.0077688

**Published:** 2013-10-17

**Authors:** Miguel Angel Tola-Arribas, María Isabel Yugueros, María José Garea, Fernando Ortega-Valín, Ana Cerón-Fernández, Beatriz Fernández-Malvido, Antonio San José-Gallegos, Marta González-Touya, Ana Botrán-Velicia, Vanessa Iglesias-Rodríguez, Bárbara Díaz-Gómez

**Affiliations:** 1 Department of Neurology, Hospital Universitario Río Hortega, Valladolid, Spain; 2 Department of Geriatrics, Hospital Universitario Río Hortega, Valladolid, Spain; 3 Department of Psychology, Hospital Universitario Río Hortega, Valladolid, Spain; 4 Emergency Department, Hospital Medina del Campo, Valladolid, Spain; 5 Atención Primaria Area Oeste, Valladolid, Spain; 6 Centro de Salud Arroyo de la Vega, Alcobendas, Madrid, Spain; University Medical Center Rotterdam, Netherlands

## Abstract

**Objective:**

To describe the prevalence of dementia and subtypes in a general elderly population in northwestern Spain and to analyze the influence of socio-demographic factors.

**Methods:**

Cross-sectional, two-phase, door-to-door, population-based study. A total of 870 individuals from a rural region and 2,119 individuals from an urban region of Valladolid, Spain, were involved. The seven-minute screen neurocognitive battery was used in the screening phase. A control group was included.

**Results:**

A total of 2,170 individuals aged 65 to 104 years (57% women) were assessed. There were 184 subjects diagnosed with dementia. The crude prevalence was 8.5% (95% CI: 7.3-9.7). Age- and sex-adjusted prevalence was 5.5 (95% CI: 4.5-6.5). Main subtypes of dementia were: Alzheimer’s disease (AD) 77.7%, Lewy Body disease, 7.6% and vascular dementia (VD) 5.9%. Crude prevalences were 6.6% (AD), 0.6% (Lewy Body disease), and 0.5% (VD). Dementia was associated with age (OR 1.14 for 1-year increase in age), female sex (OR 1.79) and the absence of formal education (OR 2.53 compared to subjects with primary education or more).

**Conclusion:**

The prevalence of dementia in the study population was lower than the most recent estimates for Western Europe. There was a high proportion of AD among all dementia cases and very low prevalence of VD. Old age, female sex, and low education level were independent risk factors for dementia and AD.

## Introduction

In 2012, the European Commission published predicted population changes in European Union countries for the following 50 years [1]. It was projected that the percentage of people aged 65 years or more will increase from 17% to 30%, and the percentage of people over 80 will increase from 5% to 12%. This effect is likely to be particularly relevant to Spain, where female life expectancy already reaches 85 years, which is the highest in Europe [2]. In this context, neurological diseases, especially dementia, pose a challenge for healthcare systems worldwide [3]. For 2010, the overall cost of dementia was estimated at 210 billion USD in Western Europe alone [4]. 

The most recent systematic review of worldwide dementia prevalence and future projections was published in 2013 [5]. The 2010 estimate for Western Europe (7.3% prevalence in individuals aged 60 years or more) represents a considerable increase compared to the 2005 Delphi consensus [6]. These projections assume that disease prevalence will remain stable over time, which can greatly limit their validity [7]. These estimates can be substantially altered by a better control of vascular risk factors [8] or by the emergence of treatments that can alter the course of the disease, slow its progress, or increase survival rates. Moreover, study methods themselves can potentially introduce important sources of variability in the prevalence rates [9]. Therefore, it is necessary to monitor the epidemiology of dementia in different parts of the world. In that respect, the decrease in the number of studies on dementia prevalence in developed countries since 1990 is alarming [5]. 

Several population-based studies on dementia have been conducted in Spain over the last two decades. These studies have shown varying prevalence rates, which are largely due to methodological differences [10,11]. For the most part, research has focused on the prevalence of Alzheimer’s disease (AD) and vascular dementia (VD). The prevalences of other primary dementias, such as dementia with Lewy bodies (DLB) or frontotemporal dementia (FTD), have barely been addressed [12,13]. In 2009, Virués et al. estimated the age- and sex-adjusted dementia prevalence to be 7.5% in Spain among individuals aged ≥ 75 years. They analyzed nine Spanish population samples, which were obtained from the population of survivors who participated in prior population-based studies [14].

The DEMINVALL project is an epidemiological study of dementia conducted in the province of Valladolid, northwestern Spain. Its main objectives are to 1) describe the prevalence of dementia and its subtypes; 2) identify the frequency, characteristics, and determining factors of undiagnosed dementia in the community; and 3) assess the effects of nutritional status and diet characteristics on dementia. In this report, we present the results of the prevalence study and analyze the effects of age, gender, education level, and place of residence (rural or urban setting) on dementia prevalence.

## Materials and Methods

DEMINVALL is a cross-sectional, two-phase, door-to-door, population-based study. The prevalence date was February 1, 2009. To be eligible, individuals must have been aged ≥ 65 years on the prevalence date and must have lived at least 6 months of the previous year in the selected geographic area. A detailed description of the study methods, main demographic and sociocultural findings, and analysis of the participant attrition can be found elsewhere [15]. 

### Study population

A mixed urban and rural population was selected. Community-dwelling and nursing home residents were included. The rural population was composed of subjects aged ≥ 65 years of 11 townships west of the Valladolid province (870 residents). This region is characterized by low population density and lower socioeconomic status among individuals belonging to this age category. The urban population was selected from subjects aged ≥ 65 years of the Parquesol and Campo Grande health districts in the city of Valladolid (6,183 residents). Due to the large population size, the survey was based on a 34.2%, random, 5-year, age- and sex-stratified sample comprising 2,119 individuals. The size was calculated in order to provide an estimated 6.5% prevalence, 1% precision, 95% confidence interval (CI), and 20% expected losses. Participants were selected from the registry of Social Security health card holders, which provides virtually universal coverage for the population in this province. The data were provided by the local healthcare authorities and had been updated most recently on November 1, 2008. On this date, the immigration rate in this portion of the population was below 1% for the province.

### Phase 1: Screening

The screening phase was conducted between February 2009 and February 2010. The study was extensively advertised to increase participation, and an information letter was sent to all of the selected participants. Screening was conducted by 27 primary care doctors and 1 geriatrician, all of whom were trained specifically for the study protocol. Screening interviews were conducted at healthcare centers, rural doctors’ offices, or at participants’ homes. A structured questionnaire was administered to collect information regarding medical, educational, sociodemographic, employment, and lifestyle histories. The detection of dementia in primary care and the existence of prior consultations for cognitive impairment, complaint symptoms and level of care in which they were held were analyzed. All patients with a prior diagnosis of dementia were evaluated during the second phase of the study. 

The Spanish version of the 7-Minute Screen Neurocognitive Battery (7MS) was used as a screening tool [16]. A total score below 20 (8th percentile) was considered positive. This scoring system was chosen due to its higher discriminatory power in the validation of the Spanish version of the test [17,18] and because it is the cutoff point that is customarily used in clinical practice in Spain. Whenever administration of the 7MS was not possible, the abbreviated Spanish version of the Informant Questionnaire on Cognitive Decline in the Elderly (IQCODE) [19] was employed. A score of 57 or higher was considered positive. In cases of death after the prevalence date, collaboration of a close relative was requested, and a Spanish version of the Kawas Dementia Questionnaire [20] was administered. This instrument provides a structural review of the general diagnostic criteria of dementia and subtypes. 

### Phase 2: Diagnostic confirmation

The second phase was conducted by four neurologists, one geriatrician, and three neuropsychologists between May 2009 and May 2010. Participants were evaluated via external consultation with the neurology, geriatric, and clinical psychology departments of the Río Hortega University Hospital. When necessary, participants were evaluated at their homes. Comprehensive neurological and neuropsychological assessments were offered to all individuals who screened positive. In addition, a randomly selected control group of 160 subjects was included (8.3% of those who screened negative). The proportions of sex and age stratification with regard to those who screened positive were respected. A systematic laboratory study (including ApoE genotyping) and brain imaging tests (cranial CT or 1.5T MRI) were performed. For cases of parkinsonism-related dementia, a single-photon emission computed tomography imaging study with123 FP-CIT (DaTSCAN; GE Healthcare) was performed.

The neuropsychological evaluation consisted of the Spanish version of the Cambridge Examination of Mental Disorders of the Elderly [21]. This instrument includes a cognitive assessment (Cambridge Cognitive Examination), functional and neuropsychiatric assessments, and an informant interview. Disabilities were evaluated using the Rapid Disability Rating Scale-2 [22], and the severity of dementia was assessed using the Clinical Dementia Rating (CDR) [23]. 

Clinical diagnosis of dementia and subtype was established by consensus between the researchers involved in phase 2. The post-mortem dementia questionnaires were evaluated by the principal investigator. The DSM-IV criteria [24] were applied for the diagnosis of dementia syndrome. Additionally, the NINCDS-ADRDA criteria for AD [25], the NINDS-AIREN criteria for VD [26], and the Neary criteria for FTD [27] were used to classify dementia subtypes. Cases of dementia associated with parkinsonism were grouped under the denomination of Lewy body disease (LBdis), including DLB, according to McKeith’s criteria [28], and Parkinson’s disease-associated dementia (PDD). Presentation of parkinsonism at least one year prior to dementia was the temporal criteria used to diagnose PDD. AD with cerebrovascular disease was regarded as present in those patients that met the criteria of NINCDS-ADRDA for possible AD and who presented significant levels of small vessel ischemic changes, strategic lacunar or large vessel infarcts on brain imaging and, in addition, who had a history of stroke or impaired neurological examination (focal deficit or gait disturbances). Secondary dementia was classified as having an identifiable cause. When the clinical information was insufficient to reach an etiologic classification, undetermined dementia was diagnosed.

### Ethics Statement

This study was approved by the Ethics and Clinical Investigation Committee of the Rio Hortega University Hospital of Valladolid (Comité Ético de Investigación Clínica del Hospital Universitario Río Hortega de Valladolid) and the Regional Health Management Office of Castilla y León (SACYL). All participants provided written informed consent prior to participation. Their capacity to consent was judged by experienced physicians before starting the interview, thus clinician-based. In cases where capacity to consent was doubted, informed consent was obtained by next of kin, care takers as well as guardians on the behalf of participants. Clinical follow-up was offered to all patients with undetected dementia. All other non-neurological processes detected were reported to the primary care physicians. Appropriate measures were taken to ensure confidentiality of the data. The database was inscribed in the Spanish Agency for Data Protection.

### Statistical analyses

Point prevalence was used to measure disease frequency. Non-responders (non-located and refusals) were excluded from the prevalence calculation. Age- and sex-specific prevalence were calculated in 5-year intervals, from ≥ 65 to ≥ 90 years, with a 95% CI based on a binomial distribution. Age-adjusted and age- and sex-adjusted prevalence of dementia was obtained. European standard population weights were used with the following weightings: 0.36, 0.27, 0.18, 0.09, and 0.09 for ages 65-69, 70-74, 75-79, 80-84, and ≥85, respectively, and 0.5 for both males and females. Pearson’s chi-squared test was used to evaluate differences between categorical variables. A multivariate analysis with logistic regression was used to assess the association between risk factors, dementia, and AD. Age was introduced as a continuous variable. Adjusted odds ratios (OR) were calculated with 95% CI. In all cases, the significance level was set at < 0.05. SPSS software version 15.0 (SPSS, Inc., Chicago, IL) was used for all statistical analyses. 

## Results

**Figure 1:**
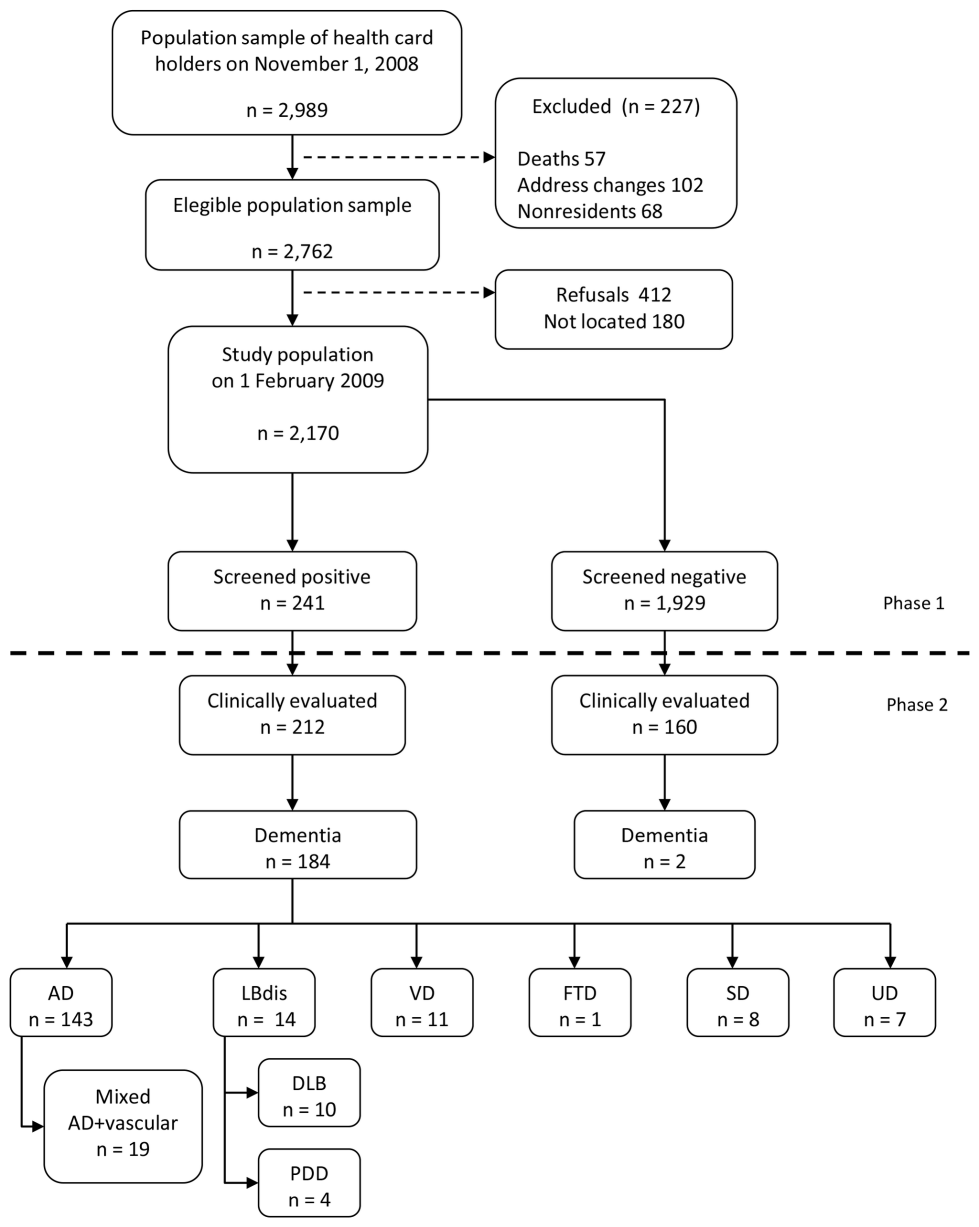
Study flow chart with results from the screening and diagnostic phases. AD: Alzheimer’s disease. LBdis: Lewy body disease. DLB: Dementia with Lewy bodies. PDD: Parkinson´s disease-associated dementia. VD: Vascular dementia. FTD: Fronto-temporal dementia . SD: Secondary dementia. UD: Undetermined dementia.

Population attrition and the results of the clinical work-up are depicted in Figure 1. Out of the 2,989 individuals initially selected, 227 (7.6%) were excluded for not satisfying the inclusion criteria. The participation rate among eligible subjects was 79%. A total of 2,170 individuals aged between 65 and 104 years (mean age: 76.5 ± 7.8 years; 57% females; 5.2% institutionalized) were screened. Among these, 1,459 subjects were from the urban area and 711 from the rural area. 

Table 1 presents the education levels of the participants who provided this information (n=2,118). The percentage of illiterate or subjects without formal education was significantly higher in women than in men (24.6% vs. 19.8%, p<0.01), as well as in rural areas than in urban areas (42.1% vs. 13%, p<0.001). 

**Table 1:** Participant education levels organized by living environment and gender.

	**Urban population (n=1,425)**		**Rural population (n=693)**		**Total population (n=2,118)**	
**Education level**	**Men, n (%)**	**Women, n (%)**	**Men, n (%**)	**Women, n (%)**	**Men, n (%)**	**Women, n (%)**
Illiterate	1 (0,2%)	7 (0,8%)	4 (1,2%)	13 (3,7%)	5 (0,5%)	20 (1,7%)
Less than primary school	50 (8,7%)	127 (14,9%)	126 (36,8%)	148 (42,2%)	176 (19,2%)	275 (22,9%)
Primary school	247 (43,1%)	472 (55,4%)	196 (57,3%)	178 (50,7%)	443 (48,4%)	650 (54,0%)
Secondary school and higher	275 (48,0%)	246 (28,9%)	16 (4,7%)	12 (3,4%)	291 (31,8%)	258 (21,4%)
Total	573 (100%)	852 (100%)	342 (100%)	351 (100%)	915 (100%)	1,203 (100%)

A total of 241 (11%) subjects had positive screenings. Of these, 212 (88%) were assessed during the second phase, in which 184 dementia cases were identified (mean age: 84.1 ± 7.1 years; 75% females; 32.1% institutionalized). There was a previous diagnosis of dementia in 83 subjects (45.1%) and a family history of dementia in at least one first-degree relative in 28 subjects (15.2% of the total of identified cases). Two false-negatives (one AD and one VD) were found in the control group. Dementia subtypes and their frequencies were as follows: AD 143 (77.7%), LBdis 14 (7.6%), VD 11 (6%), FTD 1 (0.5%), secondary dementias 8 (4.3%), and undetermined dementia 7 (3.8%). In the AD group, 19 patients had associated cerebrovascular disease. Of the 14 individuals in the LBdis group, 10 were classified as DLB and 4 as PDD. In the secondary dementia group, the following were identified: three cases of disseminated neoplasia with brain involvement, two cases of normal pressure hydrocephalus, two related to alcohol abuse, and one of anoxic encephalopathy due to cardiac arrest. 

According to the CDR, 30.4% had mild dementia, 29.9% moderate dementia, and 39.7% advanced dementia. Neuroimaging tests were conducted in 125 (68%) of the 184 dementia patients. APOE genotyping was conducted in 123 cases; 38% were APOE ε4-positive. This percentage was 42.7% for AD (APOE ε4-positive in 41 of 96 AD patients who underwent this test). Among the 83 patients with previously diagnosed dementia, 21 (25.3%) were under treatment with acetylcholinestarase inhibitors, 12 with memantine (14.5%) and 3 (3.6%) in combined therapy. As a whole, 36 patients out of the total of 184 (19.6%) identified in the study were receiving disease modifying drugs. 

Table 2 shows the crude, age-adjusted and age- and sex-adjusted prevalence of dementia. The crude prevalence of overall dementia was 8.5% (95% CI: 7.3-9.7). The prevalence was greater in women than in men (11.2% vs. 4.9%, p<0.001) and in subjects with low education levels. There were no significant differences between prevalence values obtained from the rural and urban areas. The age- and sex-adjusted prevalence of dementia for subjects ≥ 75 years of age was 12.4% (95% CI: 10.5-14.3). The crude prevalence of dementia in 113 institutionalized subjects was 52.2% (95% CI: 43-61.4). 

**Table 2:** Crude, age-adjusted and age- and sex-adjusted prevalence of dementia and subtypes, organized by living environment and education level.

	**Crude prevalence**		**Age-adjusted prevalence**	**Age- and sex-adjusted prevalence**
	PR	PE (95% CI)	PE (95% CI)	PE (95% CI)
**Prevalence by dementia subtype**				
*Overall dementia*				
Men	46/933	4.9 (3.5-6.3)	4.0 (2.7-5.3)	
Women	138/1237	11.2 (9.4-12.9)	7.0 (5.6-8.4)	
Both	184/2170	8.5 (7.3-9.7)	6.0 (5.0-7.0)	5.5 (4.5-6.5)
*Alzheimer´s disease*				
Men	26/933	2.8 (1.7-3.8)	2.2 (1.3-3.1)	
Women	117/1237	9.5 (7.8-11.1)	5.8 (4.5-7.1)	
Both	143/2170	6.6 (5.5-7.6)	4.5 (3.6-5.4)	4.0 (3.2-4.8)
*Vascular dementia*				
Men	4/933	0.4 (0-0.8)	0.3 (0-0.7)	
Women	7/1237	0.6 (0.1-1.0)	0.3 (0-0.6)	
Both	11/2170	0.5 (0.2-0.8)	0.4 (0.1-0.7)	0.3 (0.1-0.5)
Lewy body disease				
Men	6/933	0.6 (0.1-1.2)	0.4 (0-0.8)	
Women	8/1237	0.6 (0.2-1.1)	0.5 (0.1-0.9)	
Both	14/2170	0.6 (0.3-1.0)	0.5 (0.2-0.8)	0.5 (0.2-0.8)
**Prevalence by residential environment**				
*Urban residential environment*				
Men	33/585	5.6 (3.8-7.5)	4.5 (2.8-6.2)	
Women	91/874	10.4 (8.4-12.4)	6.3 (4.7-7.9)	
Both	124/1459	8.5 (7.1-9.9)	5.8 (4.6-7.0)	5.4 (4.2-6.6)
*Rural residential environment*				
Men	13/348	3.7 (1.7-5.7)	3.1 (1.3-4.9)	
Women	47/363	12.9 (9.5-16.4)	8.8 (5.9-11.7)	
Both	60/711	8.4 (6.4-10.5)	6.3 (4.5-8.1)	5.9 (4.2-7.6)
**Prevalence by education level**				
*Illiterate*	8/25	32.0 (13.7-50.3)	23.1 (6.6-39.7)	28.7 (11.0-46.5)
*Less than primary school*	51/451	11.3 (8.4-14.2)	7.8 (5.3-10.2)	7.3 (4.9-9.7)
*Primary school*	86/1093	7.9 (6.3-9.5)	5.3 (4.0-6.6)	4.9 (3.6-6.1)
*Secondary school and higher*	20/549	3.6 (2.1-5.2)	3.1 (1.7-4.6)	2.8 (1.1-4.1)

PR: Crude prevalence ratio (cases divided by population). PE: Point prevalence estimation. CI: Confidence interval.

Table 3 shows the age- and sex-specific prevalences of the main dementia subtypes. The frequency of dementia and AD was below 2% of individuals of each gender aged 65 to 75 years. The frequency of dementia and AD clearly increased in individuals aged ≥ 75 years, except in males aged ≥ 85 years. Table 4 details the influence of various sociodemographic variables on dementia and AD. The adjusted OR of the logistic regression analysis are presented. A strong association was found between age, female gender, and low education level for both dementia and AD. 

**Table 3:** Age- and sex-specific counts, showing crude prevalence of dementia and its main subtypes.

**Age group**		**Population**	**Dementia**		**AD**		**LBdis**		**VD**	
			Cases	P,% (95% IC)	Cases	P,% (95% IC)	Cases	P,% (95% IC)	Cases	P,% (95% IC)
**Men**										
65-69		253	3	1.2 (0-2.5)	1	0.4 (0-1.2)	0	---	1	0.4 (0-1.2)
70-74		238	4	1.7 (0-3.3)	2	0.8 (0-2.0)	0	---	0	---
75-79		203	12	5.9 (2.7-9.2)	6	3.0 (0.6-5.3)	1	0.5 (0-1.5)	0	---
80-84		144	18	12.5 (7.1-17.9)	10	6.9 (2.8-11.1)	4	2.8 (0.1-5.5)	3	2.1 (0-4.4)
85-89		68	5	7.4 (1.1-13.6)	4	5.9 (0.3-11.5)	1	1.5 (0-4.3)	0	---
≥ 90		27	4	14.8 (1.4-28.2)	3	11.1 (0-23.0)	0	---	0	---
**Women**										
65-69		296	5	1.7 (0.2-3.2)	3	1.0 (0-2.2)	2	0.7 (0-1.6)	0	---
70-74		237	4	1.7 (0-3.3)	4	1.7 (0-3.3)	0	---	0	---
75-79		246	25	10.2 (6.4-13.9)	21	8.5 (5.0-12.0)	1	0.4 (0-1.2)	2	0.8 (0-1.9)
80-84		220	31	14.1 (9.5-18.7)	26	11.8 (7.6-16.1)	1	0.5 (0-1.3)	2	0.9 (0-2.2)
85-89		151	41	27.2 (20.1-34.2)	33	21.9 (15.3-28.4)	4	2.6 (0.1-5.2)	1	0.7 (0-2.0)
≥ 90		87	32	36.8 (26.6-46.9)	30	34.5 (24.5-44.5)	0	---	2	2.3 (0-5.4)
**Men and women**										
65-69		549	8	1.5 (0.5-2.5)	4	0.7 (0-1.4)	2	0.4 (0-0.9)	1	0.2 (0-0.5)
70-74		475	8	1.7 (0.5-2.8)	6	1.3 (0.3-2.3)	0	---	0	---
75-79		449	37	8.2 (5.7-10.8)	27	6.0 (3.8-8.2)	2	0.4 (0-1.1)	2	0.4 (0-1.1)
80-84		364	49	13.5 (10.0-17.0)	36	9.9 (6.8-13.0)	5	1.4 (0.2-2.6)	5	1.4 (0.2-2.6)
85-89		219	46	21.0 (15.6-26.4)	37	16.9 (11.9-21.9)	5	2.3 (0.3-4.3)	1	0.5 (0-1.3)
≥ 90		114	36	31.6 (23.0-40.1)	33	28.9 (20.6-37.3)	0	---	2	1.8 (0-4.2)

AD: Alzheimer’s disease. LBdis: Lewy body disease. VD: Vascular dementia. P: Prevalence.

**Table 4:** Relation between sociodemographic factors, dementia, and Alzheimer’s disease.

**Characteristics**	**Dementia, OR (IC 95%)**	**Alzheimer´s disease, OR (IC 95%)**
Age, years*	1.14 (1.12-1.17)	1.15 (1.12-1.18)
**Gender, women**	1.79 (1.22-2.63)	2.57 (1.60-4.11)
**Education**		
Illiterate	9.69 (3.45-27.24)	6.58 (1.99-21.72)
Less than primary school	2.53 (1.45-4.44)	2.65 (1.38-5.11)
Primary school	1.94 (1.15-3.27)	2.21 (1.20-4.08)
**Residence in rural area**	0.99 (0.72-1.37)	1.14 (0.80-1.63)

Adjusted odds ratio (OR) for all variables included in the table; CI, confidence interval.

Education level reference category: secondary education and higher.

* Age as a continuous variable. OR for one-year age increments

## Discussion

The DEMINVALL study offers good global quality parameters according to quality criteria proposed to assess studies of dementia prevalence based on the sample size, study design, inclusion of a control group, degree of participation, and diagnostic procedures [5]. After adjusting for age and sex, the prevalence of dementia and AD in our population fell into an intermediate-low range compared to previously published rates in other Spanish [10] and Western European populations [5,29,30]. We found a considerable preponderance of AD, very low frequency of VD, and low prevalence of dementia in subjects aged ≤ 75 years. The age- and sex-adjusted dementia prevalence was clearly higher (12.4% vs. 7.5% in subjects aged ≥ 75 years) compared to a recent multicenter Spanish study [14]. Therefore, the large number of dementia patients in our region seems to be highly influenced by population aging and the particularly increased life expectancy of women. Table 5 shows the crude prevalences of dementia, AD and VD of some population-based surveys in southern Europe within the last few years. The most recent that include the prevalence of dementia and its subtypes have been selected [11,12,31-34].

**Table 5:** Crude prevalence rates of dementia, Alzheimer´s disease and vascular dementia in Southern Europe and in the present study.

**Survey area**	**No. individuals**	**Age (year)**	**Year of survey**	**Dementia, P (%)**	**AD, P (%)**	**VD, P (%)**
Buttapietra, Italy	222	≥ 75	1996	15.8	6.7	3.6
Conselice, Italy	1016	≥ 65	1999	5.9	3.0	2.7
Tuscany, Italy	1600	≥ 65	2000	6.2	4.2	1.4
El Prat de Llobregat, Spain	1754	≥ 70	2002	9.4	6.5	1.2
Murcia, Spain	1074	≥ 65	2003	5.5	4.5	0.8
Sicily, Italy	280	≥ 60	2005	7.1	4.1	1.1
Valladolid, Spain	2170	≥ 65	2009	8.5	6.6	0.5

AD: Alzheimer’s disease. VD: Vascular dementia. P: Prevalence.

The age- and sex-adjusted prevalence rates were higher in the rural population than in the urban population, but this difference was not significant. Both the higher proportion of women in the urban area (60% vs. 52%) and the lower educational level in the rural area could have contributed to balancing out possible prevalence differences that have been previously reported in other studies, with higher prevalence rates in rural areas, especially in AD [35].

We analyzed the frequency of dementia associated with parkinsonism as a group termed LBdis, using a temporary criterion to differentiate between DLB and PDD. Although the literature on this disease is limited, our results support those reported in other studies [36,37]. We found a crude prevalence of 0.6%, which represented 7.6% of all dementia cases and was the second-most prevalent subtype after AD. In Spain, studies conducted in El Prat de LLobregat and Munguialde have reported similar numbers for LBdis prevalence, although lower than VD [12,13]. 

The most striking finding of the DEMINVALL study is the low prevalence of VD, which is the lowest ever described in Spain [10,14] and one of the lowest described in Europe [38]. Some aspects of the study methods may have contributed to this result. First, the screening method, 7MS, was originally designed as an AD detection test, although it has proven useful for detecting other types of dementia [39]. The 7MS is a highly sensitive and specific screening battery that primarily focuses on episodic memory deficiencies; this may have contributed to the high degree of AD detection but could be less sensitive to detect VD. Nonetheless, this screening battery includes tests to assess frontal executive functions that psychometrically exhibit good sensitivity for detecting subcortical vascular dementia [16]. Nevertheless, the number of false-negatives in the control group was small. However, one case of VD was included in the control group; this raises the possibility that other, undetected cases existed. Second, many subjects with dementia (32%) were not able to undergo neuroimaging tests to confirm the presence of vascular lesions of little clinical relevance. This fact is a major limitation of most population-based studies. Finally, the diagnostic criteria for the different subtypes of dementia, especially VD, impose artificial constraints that neuropathological studies have proven to be nonexistent [40] and for which no broad consensus currently exists. In our case, we used a restrictive criterion to diagnose AD with associated cerebrovascular disease, which required the simultaneous presence of clinical and neuroimaging findings. Consequently, fewer than 15% of the AD cases met the criteria for mixed dementia. Several patients could have also been included in the possible VD category if clinical criteria had been strictly enforced, as reported in the study conducted in El Prat de Llobregat, Spain [12]. Even after considering these factors, which may limit the validity of the obtained results, we believe that the low prevalence of “pure” VD is a real result. The frequency of vascular risk factors in our population [15] is similar to Southern European countries; reduced vascular mortality has been confirmed in these regions [41]. Furthermore, it is possible that the Mediterranean diet, which is unique to our region, has a great impact on cardiovascular health, especially with respect to the decrease in the incidence of stroke and, consequently, VD [42,43].

Old age, low education level, and female sex were strongly associated with both dementia and AD. These factors have been previously identified, to some extent, as dementia risk factors in developed countries [30,44]. In Spain, some authors have found no correlation between dementia and sex [11] or years of education [45]. The effect of age on male subjects in our study was much less striking, similar to previous reports in our country [10]. This could be related with a lower dementia incidence [46] and a lower disease survival in men [47].

The main limitation of the present study, as with most epidemiological population-based studies, is participation bias. Of 2,989 individuals who were initially identified, 2,170 were finally evaluated (79% of all eligible ones). The characteristics of non-responders were similar to those of the evaluated group, although greater compliance was observed in the rural area [15]. A 20% loss to follow-up was estimated in the calculation of the urban sample to reduce this bias. Furthermore, even though these results could be useful for estimating dementia cases in our geographic region, they are hard to extrapolate to the entire Spanish population given its cultural, demographic, and social diversity. 

In conclusion, we present dementia prevalence rates in a population from northwestern Spain. These numbers are lower than those that have been reported for neighboring countries. In particular, the very low prevalence of VD and the high proportion of AD among all dementia cases are striking. The results from future epidemiological studies in Spain and in Western Europe will allow for the determination of whether our findings are sporadic or if these trends in dementia truly exist, especially for VD.
